# *TRIM45* functions as a tumor suppressor in the brain via its E3 ligase activity by stabilizing p53 through K63-linked ubiquitination

**DOI:** 10.1038/cddis.2017.149

**Published:** 2017-05-25

**Authors:** Jindong Zhang, Chuanxia Zhang, Jun Cui, Jiayu Ou, Jing Han, Yunfei Qin, Feng Zhi, Rong-Fu Wang

**Affiliations:** 1 Zhongshan School of Medicine, Sun Yat-sen University, Guangzhou 510080, People's Republic of China; 2 Collaborative Innovation Center of Cancer Medicine, Sun Yat-sen University, Guangzhou 510080, People's Republic of China; 3 Key Laboratory of Gene Engineering of the Ministry of Education, State Key Laboratory of Biocontrol, School of Life Sciences, Sun Yat-sen University, Guangzhou 510006, People's Republic of China; 4 Modern Medical Research Center, Third Affiliated Hospital of Soochow University, Changzhou 213000, People's Republic of China; 5 Center for Inflammation and Epigenetics, Houston Methodist Research Institute, 6670 Betner Avenue, Houston, TX 77096, USA

## Abstract

Tripartite motif-containing protein 45 (TRIM45) belongs to a large family of RING-finger-containing E3 ligases, which are highly expressed in the brain. However, little is known regarding the role of *TRIM45* in cancer biology, especially in human glioma. Here, we report that *TRIM45* expression is significantly reduced in glioma tissue samples. Overexpression of *TRIM45* suppresses proliferation and tumorigenicity in glioblastoma cells *in vitro* and *in vivo*. In addition, CRISPR/Cas9-mediated knockout of *TRIM45* promotes proliferation and inhibits apoptosis in glioblastoma cells. Further mechanistic analyses show that *TRIM45* interacts with and stabilizes p53. *TRIM45* conjugates K63-linked polyubiquitin chain to the C-terminal six lysine residues of p53, thereby inhibiting the availability of these residues to the K48-linked polyubiquitination that targets p53 for degradation. These findings suggest that *TRIM45* is a novel tumor suppressor that stabilizes and activates p53 in glioma.

Gliomas are the most common type of brain tumors and account for ~80% of primary malignant tumors in the central nervous system (CNS).^1, 2, 3^ Currently, the standard treatment strategies for glioma are surgical resection and adjuvant chemotherapy with temozolomide (TMZ) combined with radiotherapy.^4^ However, patients with malignant glioma, especially those with glioblastoma (GBM), have little benefit from standard treatments, due to the tumor’s malignant features such as rapid cell proliferation, robust invasiveness, and increased angiogenesis, and lack of specific therapeutic targets for immunotherapy.^1, 5^ Therefore, it is imperative to determine the mechanisms underlying glioma tumorigenesis and to develop more effective therapeutic strategies.

The tumor suppressor p53 is a crucial cellular stress sensor that triggers cell-cycle arrest, apoptosis, and senescence in response to many diverse stresses, including DNA damage, hyperproliferative signals, hypoxia, oxidative stress, ribonucleotide depletion, and nutrient starvation.^6^ p53-mediated cellular responses primarily depend on the function of p53 as a transcriptional activator and on the p53-mediated induction of particular target genes.^6^ As even minimal changes in p53 expression can be deleterious to the organism, tight regulation of p53 is essential in normal cells.^7, 8, 9^ A plethora of redundant post-transcriptional modifications can occur in p53, including ubiquitination, acetylation, phosphorylation, methylation, neddylation, and sumolytion, all of which significantly affect its activity and function.^7^

Ubiquitination is a reversible post-translational modification that either targets proteins for degradation or regulates protein function.^10^ Ubiquitin itself contains seven lysines, and each of these can be further conjugated to another ubiquitin molecule at its carboxyl terminus to form different types of polyubiquitin chains. The lysine-(K) 48- and K63-linked polyubiquitin chains are the predominant types of ubiquitin linkage.^11^ K48-linked polyubiquitination targets proteins for proteasomal degradation, and K63-linked polyubiquitination has a role in cell signaling. The effects and mechanisms of several E3 ligases on p53 have been reported.^12^ The RING-finger E3 ligase mouse double minute homolog 2 (MDM2) conjugates K48-linked polyubiquitin chains to p53, and this modification targets p53 for proteasomal degradation.^13, 14^

The tripartite motif-containing (TRIM) family is characterized by several unique structural motifs: a RING-finger domain, one or two B-box domain and a coiled-coil domain.^15, 16^ Most TRIM proteins possess a variable C-terminal domain, which has a role in substrate binding.^17^ As a member of the TRIM family, *TRIM45* contains a filamin-type immunoglobulin (FLMN) domain in its carboxy-terminal region. *TRIM45* was reported to negatively regulate the MAPK signaling pathway by inhibiting RACK1/PKC complex formation.^18, 19^ It also negatively regulates NF-*κ*B signaling.^20^ Although *TRIM45* mRNA is highly expressed in the brain of human adult and embryonic tissues,^18^ its function in primary CNS tumors has not been investigated yet.

Here we report that *TRIM45* expression is reduced in glioma tissues, and *TRIM45* suppresses proliferation and tumorigenicity in GBM cells *in vivo* and *in vitr*o. Furthermore, *TRIM45* interacts with and stabilizes p53 by promoting K63-linked polyubiquitination of p53, thereby inhibiting the subsequent K48-linked polyubiquitination that targets p53 for degradation. These findings suggest that *TRIM45* functions as a novel regulator responsible for maintaining p53 stability in glioma.

## Results

### *TRIM45* is downregulated in primary gliomas

**Figure 1:**
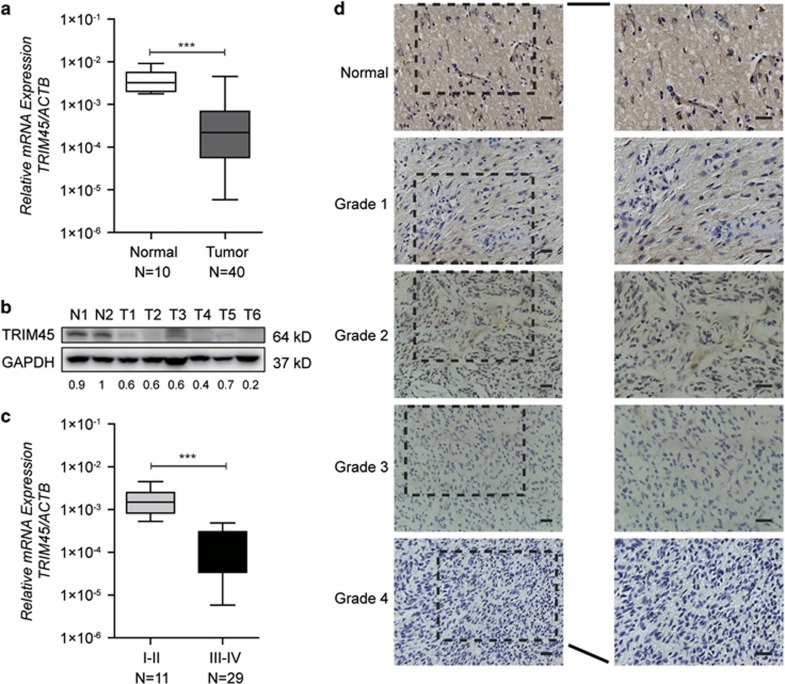
TRIM45 expression is reduced in glioma tissues. (**a**) *TRIM45* mRNA levels in normal brain tissues (Normal) and glioma tissues (Tumor) were determined by real-time PCR. (**b**) Representative images from immunoblot analyses of *TRIM45* levels in normal brain tissues (N) and glioma tissues (T). Protein expression levels were normalized to glyceraldehyde 3-phosphate dehydrogenase (GAPDH) levels. (**c**) Comparison of *TRIM45* mRNA levels among gliomas of different pathological grades. (**d**) Representative images from immunohistochemistry (IHC) assays of paraffin-embedded specimens. Scale bars: 100 *μ*m. Data in panels (**a** and **c**) are presented as the mean±S.D. ***P<0.001

To determine the functional and clinical relevance of *TRIM45* in human glioma, we first examined *TRIM45* expression levels in normal brain tissues and human primary glioma tissues using quantitative real-time PCR (qRT-PCR) and immunoblot assays. As shown in Figure 1a, *TRIM45* mRNA was significantly downregulated in glioma samples compared with normal brain tissue samples. Similar results were obtained from immunoblotting experiments, showing the downregulation of *TRIM45* protein expression in tumor tissues (Figure 1b). We subsequently investigated whether *TRIM45* expression levels represented a distinct molecular signature for a subset of gliomas. *TRIM45* mRNA levels were lower in high-grade (WHO grade III/IV) gliomas compared with low-grade (WHO grade I/II) gliomas (Figure 1c). The reduced expression of *TRIM45* in glioma was further confirmed by immunohistochemistry staining of normal brain tissue sections and tumor tissue sections (Figure 1d). Taken together, these data indicated that *TRIM45* expression was downregulated in human glioma tissues.

### *TRIM45* regulates tumor cell growth *in vitro* and *in vivo*

**Figure 2:**
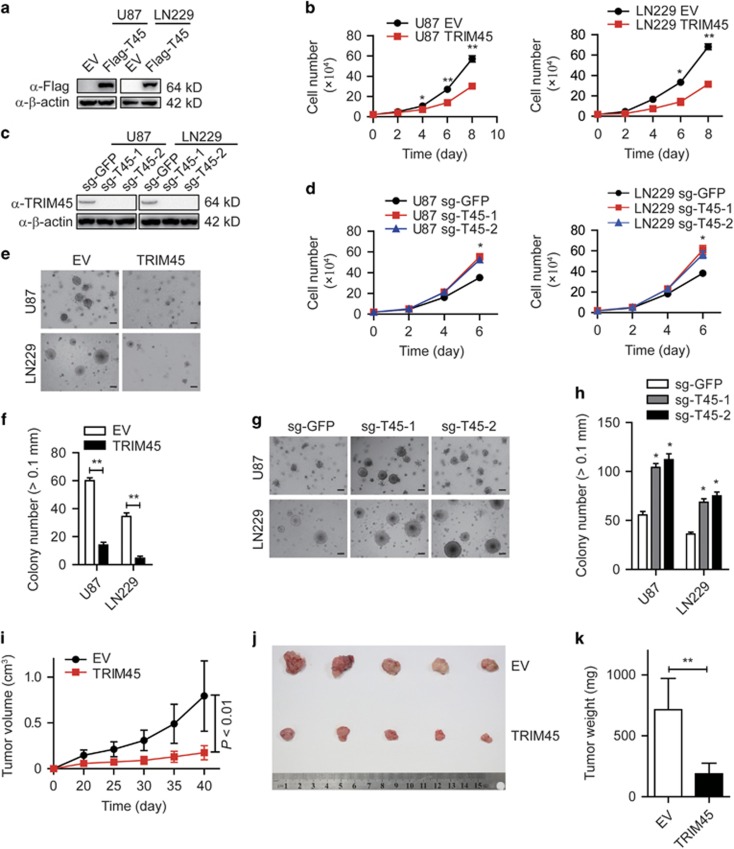
TRIM45 inhibits glioma progression *in vitro* and *in vivo.* (**a**) Lysates of TRIM45-expressing and control U87 and LN229 cells were immunoblotted with anti-Flag antibody. *β*-Actin was used as a loading control. (**c**) Lysates of *TRIM45* KO and control U87 and LN229 cells were immunoblotted with an anti-TRIM45 antibody. *β*-Actin was used as a loading control. (**b** and **d**) Growth curves were performed by plating a fixed number of cells in triplicate and counting cells at different time points. (**e**–**h**) Anchorage-independent growth assay of TRIM45-overexpressing (**e** and **f**) and *TRIM45* KO (**g** and **h**) U87 and LN229 cells. Scale bars: 200 *μ*m. (**i**–**k**) Xenograft tumors derived from LN229 cells transfected with *TRIM45* or empty vector control lentivirus. Tumor growth (**i**), representative images of tumor growth (**j**) and the measured tumor weights (**k**) are presented. Each bar represents the mean±S.D. of three independent experiments. *P<0.05 and **P<0.01

To investigate the function of *TRIM45* in the progression of glioma, we established U87 MG (referred to as U87) and LN229 glioma cell lines stably overexpressing Flag-tagged *TRIM45* (Figure 2a). The protein level of *TRIM45* in TRIM45-expressing U87 and LN229 cells was comparable to that in primary normal human astrocytes (Supplementary Figure 1). The proliferation rate was significantly inhibited in TRIM45-overexpressing U87 and LN229 cells compared with control cells (Figure 2b), whereas *TRIM45* knockdown by small hairpin RNA (shRNA) increased the proliferation rate (Supplementary Figures 2A and B). To further confirm these findings, we used the CRISPR/Cas9 system to genetically knockout (KO) the *TRIM45* gene in U87 and LN229 cells using two independent single-guide RNAs (gRNAs) targeting *TRIM45* (Figure 2c and Supplementary Figure 3). We found that *TRIM45* KO led to accelerated proliferation in both U87 and LN229 cells (Figure 2d). We next performed a colony formation assay in soft agar to evaluate the effect of *TRIM45* on anchorage-independent growth in glioma cells. Notably, overexpression of *TRIM45* significantly inhibited the number and size of the colonies in both U87 and LN229 cells, whereas KO of *TRIM45* exerted the opposite effect (Figures 2e–h). Similar results were observed in *TRIM45*-knockdown glioma cells (Supplementary Figure 4). These results suggest that *TRIM45* has an important role in the tumorigenicity of GBM cells *in vitro*.

We next evaluated the effects of *TRIM45* on tumorigenicity using xenografts mouse model. TRIM45-overexpressing tumors grew at a significantly slower rate compared with control tumors (Figure 2i). The tumors derived from TRIM45-overexpressing cells were smaller and weighed less than the control tumors (Figures 2j and k). Taken together, these data indicate that *TRIM45* inhibits GBM cell growth *in vitro* and *in vivo.*

### *TRIM45* promotes apoptosis in GBM cells

**Figure 3:**
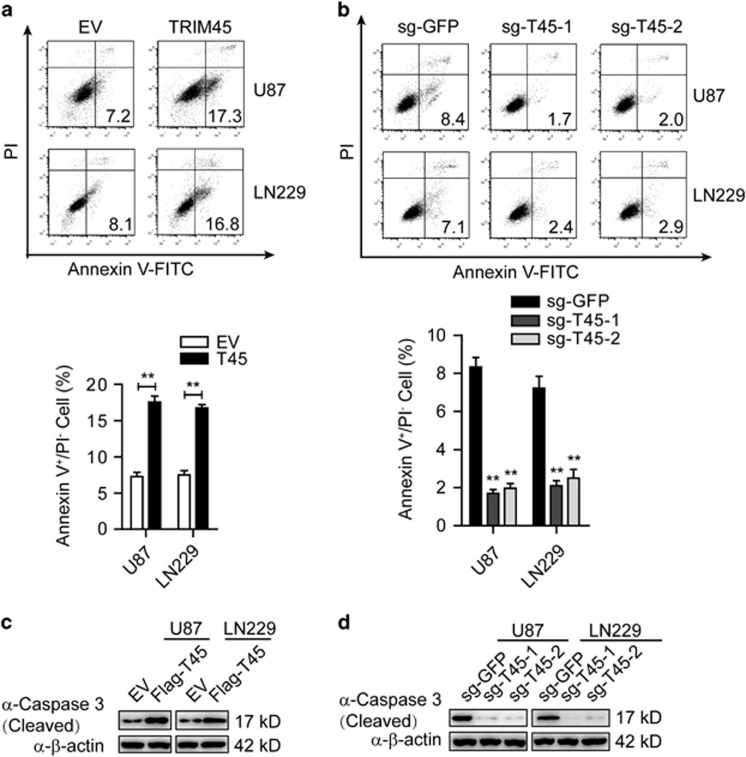
TRIM45 promotes apoptosis in GBM cells. (**a** and **b**) The TRIM45-overexpressing (**a**) and KO (**b**) U87 and LN229 cells were incubated with FITC-Annexin V solution and subjected to FACS analysis. (**c** and **d**) Lysates of TRIM45-overexpressing (**c**) and *TRIM45* KO (**d**) U87 and LN229 cells were immunoblotted with an anti-caspase-3 antibody. *β*-Actin was used as a loading control. Each bar represents the mean±S.D. of three independent experiments. **P<0.01

To further investigate the mechanism of TRIM45-mediated inhibition of GBM cell growth, we evaluated cell-cycle progression and apoptosis in GBM cells. Flow cytometry analysis demonstrated that overexpression of *TRIM45* had little effect on cell-cycle distribution (Supplementary Figure 5). Annexin V flow cytometric analysis showed that *TRIM45* overexpression markedly enhanced apoptosis (Figure 3a), and knockdown or KO of *TRIM45* inhibited apoptosis in both U87 and LN229 cells (Figure 3b and Supplementary Figure 6A). Furthermore, overexpression of *TRIM45* led to more cleaved caspase-3 (Figure 3c), whereas knockdown and KO of *TRIM45* led to less cleaved caspase-3 in both U87 and LN229 cells (Figure 3d and Supplementary Figure 6B). Taken together, these results suggest that *TRIM45* inhibits the growth of GBM cells by promoting apoptosis.

### *TRIM45* activates p53 signaling in GBM cells

We next examined the signaling pathway by which *TRIM45* inhibits proliferation and induces the apoptosis of GBM cells. As PI3K-Akt and Ras-MAPK signaling pathway are the most frequently activated signaling pathways involved in gliomagenesis and *TRIM45* was also reported to be associated with PKC-mediated modulation of the ERK-JNK and NF-*κ*B signaling pathway,^19, 20^ we examined the phosphorylation status of AKT, ERK, JNK, p38, and IKK*β* in TRIM45-overexpressing or *TRIM45* KO glioma cells. We did not detect any change in the phosphorylation status of all the kinases examined, regardless of whether the expression of *TRIM45* was upregulated or downregulated (Supplementary Figures 7A and B), suggesting that *TRIM45* does not affect the PI3K/AKT, MAPK, and NF-*κ*B signaling pathways in glioma cells.

**Figure 4:**
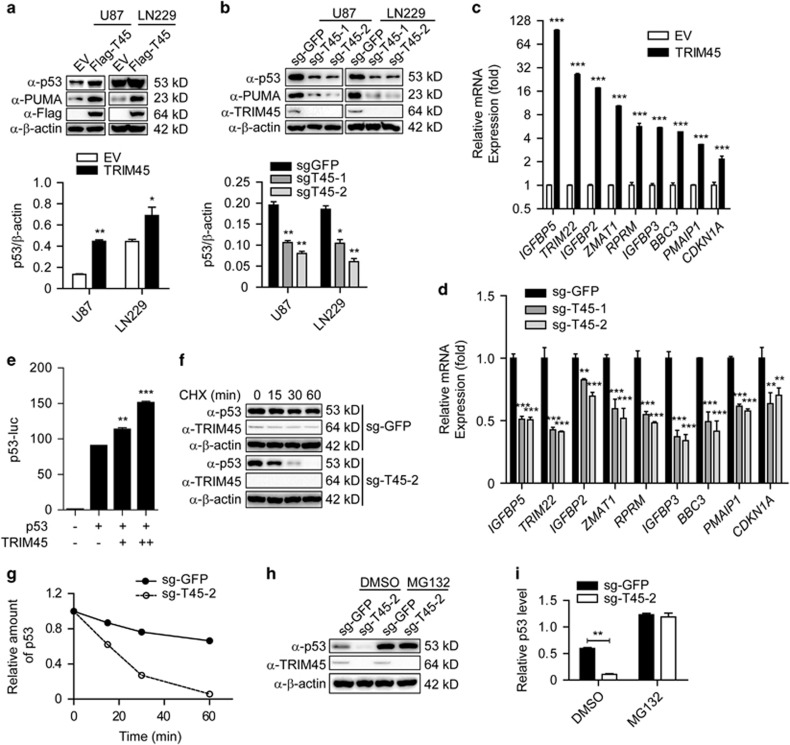
TRIM45 stabilizes and activates p53 in glioma cells. (**a**) Lysates of TRIM45-overexpressing and control U87 and LN229 cells were immunoblotted with anti-p53, anti-PUMA, and anti-Flag antibodies. *β*-Actin was used as a loading control. (**b**) Lysates of *TRIM45* KO and control U87 and LN229 cells were immunoblotted with anti-p53, anti-PUMA, and anti-TRIM45 antibodies. *β*-Actin was used as a loading control. (**c** and **d**) The mRNA levels of p53 target genes in *TRIM45* overexpression and KO U87 cells were analyzed using real-time PCR. (**e**) Luciferase activity in U87 cells transfected with p53-luc, p53, and an empty vector or a vector expressing *TRIM45* (0, 200, and 400 ng). Results are presented as reporter gene activity relative to *Renilla* luciferase activity. (**f** and **g**) Immunoblot analysis of extracts of *TRIM45* KO or control U87 cells treated for various times (as indicated above each lane) with CHX. (**h** and **i**) Lysates of *TRIM45* KO and control U87 cells treated with MG132 or dimethyl sulfoxide (DMSO) were immunoblotted with anti-p53 and anti-TRIM45 antibodies. *β*-Actin was used as a loading control. Each bar represents the mean±S.D. of three independent experiments. *P<0.05, **P<0.01, and ***P<0.001

As the p53 tumor suppressor pathway is one of the most significant pathway implicated in glioma and modulates multiple cellular processes,^21, 22^ we assessed levels of p53 in TRIM45-overexpressing and KO cells, respectively. Overexpression of *TRIM45* increased the protein levels of p53 and the p53 target gene *PUMA* in U87 and LN229 cells (Figure 4a). Conversely, *TRIM45* KO by two independent sgRNAs decreased the protein levels of p53 and PUMA in U87 and LN229 cells (Figure 4b). We further detected the differential expression of several p53 target genes using qRT-PCR in TRIM45-overexpressing cells and control cells, and we found that *TRIM45* overexpression resulted in much higher expression of p53 target genes, including *IGFBP5*, *TRIM22*, *IGFBP2*, *ZMAT1*, *RPRM*, *IGFBP3*, *BBC3* (which encodes PUMA), *PMAIP1* (which encodes NOXA), and *CDKN1A* (which encodes p21) (Figure 4c). Consistently, the mRNA levels of these genes were significantly downregulated in *TRIM45* KO cells (Figure 4d). These findings strongly suggest that *TRIM45* promotes the transcriptional activity of p53. To test this hypothesis, we evaluated the ability of *TRIM45* to influence p53 reporter activity and found that *TRIM45* significantly promoted the transcriptional activity of p53 in U87 cells (Figure 4e). Taken together, these results indicated that *TRIM45* activates the p53 signaling pathway in GBM cells.

To determine whether *TRIM45* affects apoptosis of glioma cells through p53, we knocked down p53 in TRIM45-overexpressing and control cells and examined the effect of silencing p53 on TRIM45-mediated apoptosis under TMZ treatment or without treatment (Supplementary Figure 8A). Apoptosis induced by *TRIM45* overexpression was reversed by p53 knockdown in untreated U87 cells (Supplementary Figure 8B, left). Furthermore, *TRIM45* could not enhance the TMZ-induced apoptosis in p53-knockdown U87 cells (Supplementary Figure 8B, right). These results suggest that apoptosis induced by *TRIM45* is p53-dependent.

### *TRIM45* stabilizes p53

To further determine if *TRIM45* regulates the stability of endogenous p53, we measured the half-life of p53 in the presence of cycloheximide (CHX), an inhibitor of protein synthesis. The half-life of p53 decreased in *TRIM45* KO U87 cells, indicating that *TRIM45* stabilizes endogenous p53 (Figures 4f and g). To exclude the possibility that the change in p53 protein levels occurred at the transcriptional level, we performed RT-PCR using the same glioma cells, and found that p53 mRNA level was not influenced by disruptions of *TRIM45* expression (Supplementary Figure 9). The loss of p53 induced by *TRIM45* KO was rescued by the proteasome inhibitor MG132 (Figures 4h and i), indicating that *TRIM45* protects p53 from proteasomal degradation.

### *TRIM45* interacts with p53

**Figure 5:**
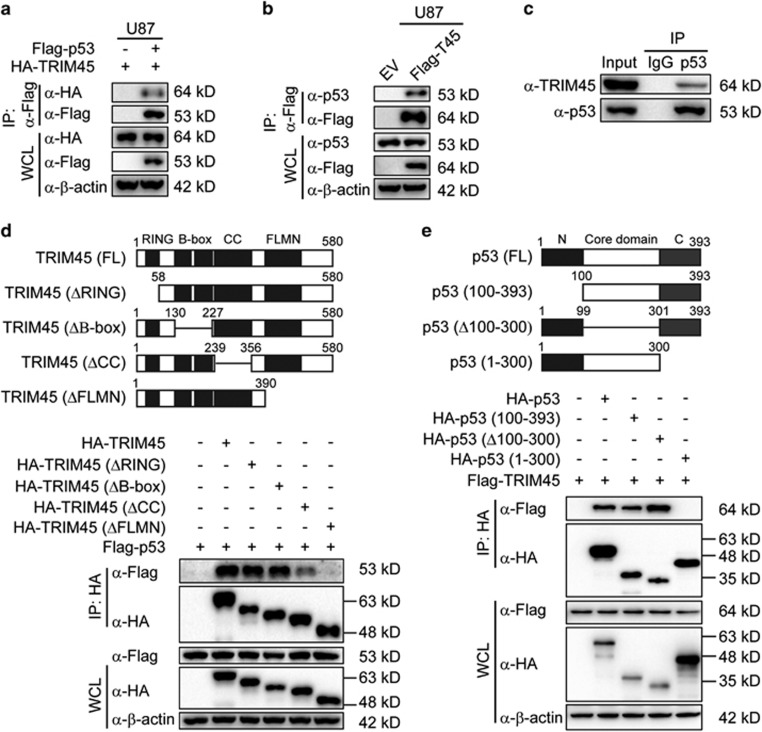
TRIM45 interacts with p53. (**a**) U87 cells were transfected with Flag-p53 and hemagglutinin (HA)-TRIM45 plasmids. The lysates were immunoprecipitated with anti-Flag beads and immunoblotted with anti-HA. (**b**) Cell extracts of TRIM45-overexpressing or control U87 cells were immunoprecipitated with anti-Flag beads and immunoblotted with the anti-p53 antibody. (**c**) U87 cell extracts were immunoprecipitated with immunoglobulin G (IgG) or the anti-p53 antibody and immunoblotted together with whole-cell lysates and the anti-TRIM45 antibody. (**d**) Top: The domain structure of TRIM45. Bottom: 293T cells were transfected with Flag-p53 and HA-TRIM45 or various HA-tagged *TRIM45* mutant constructs and treated with 10 *μ*M MG132. Whole-cell extracts were immunoprecipitated with anti-HA beads and immunoblotted with anti-Flag antibody. (**e**) Top: the domain structure of p53. Bottom: 293T cells were transfected with Flag-TRIM45 and HA-p53 or with various HA-tagged p53 mutant constructs, and treated with 10 *μ*M MG132. Whole-cell extracts were immunoprecipitated with anti-HA beads and immunoblotted with an anti-Flag antibody

The results presented in Figure 4 suggest that *TRIM45* might directly interact with p53 to activate p53 signaling. To test this hypothesis, we transfected U87 cells with HA-tagged *TRIM45* and Flag-tagged p53 expression plasmids. Co-immunoprecipitation (IP) and immunoblot experiments revealed that ectopically expressed *TRIM45* interacted with Flag-tagged p53 (Figure 5a). In addition, we found that Flag-tagged *TRIM45* interacted with endogenous p53 (Figure 5b). To further verify that *TRIM45* interacts with p53 in glioma cells, we performed IP experiments with U87 cell extracts using an anti-p53 antibody or an isotype IgG and found that *TRIM45* interacted with p53 under physiological conditions in glioma cells (Figure 5c). Taken together, these results suggest that *TRIM45* interacts with p53 in physiological conditions.

To identify the domain of *TRIM45* responsible for interaction with p53, we generated a series of deletion mutants of *TRIM45* and tested their ability to interact with p53 (Figure 5d, top). p53 exhibited a strong interaction with TRIM45-ΔRING and TRIM45-ΔB-box, a weak interaction with TRIM45-ΔCC, and no interaction with TRIM45-ΔFLMN. These results indicate that the FLMN region is essential for the interaction of *TRIM45* with p53 and that the CC region might also be required for binding to p53 (Figure 5d, bottom). We also generated three deletion mutants of p53 and evaluated their ability to bind with *TRIM45* (Figure 5e, top). We found that *TRIM45* exhibited a strong interaction with p53 (100–393) and p53 (Δ100–300) mutants, but no interaction with p53 (1–300) mutant, indicating that the C-terminal domain of p53 (301–393) is necessary for binding to *TRIM45* (Figure 5e, bottom).

### *TRIM45* mediates the K48-K63 ubiquitination transition of p53 via its E3 ligase activity

**Figure 6:**
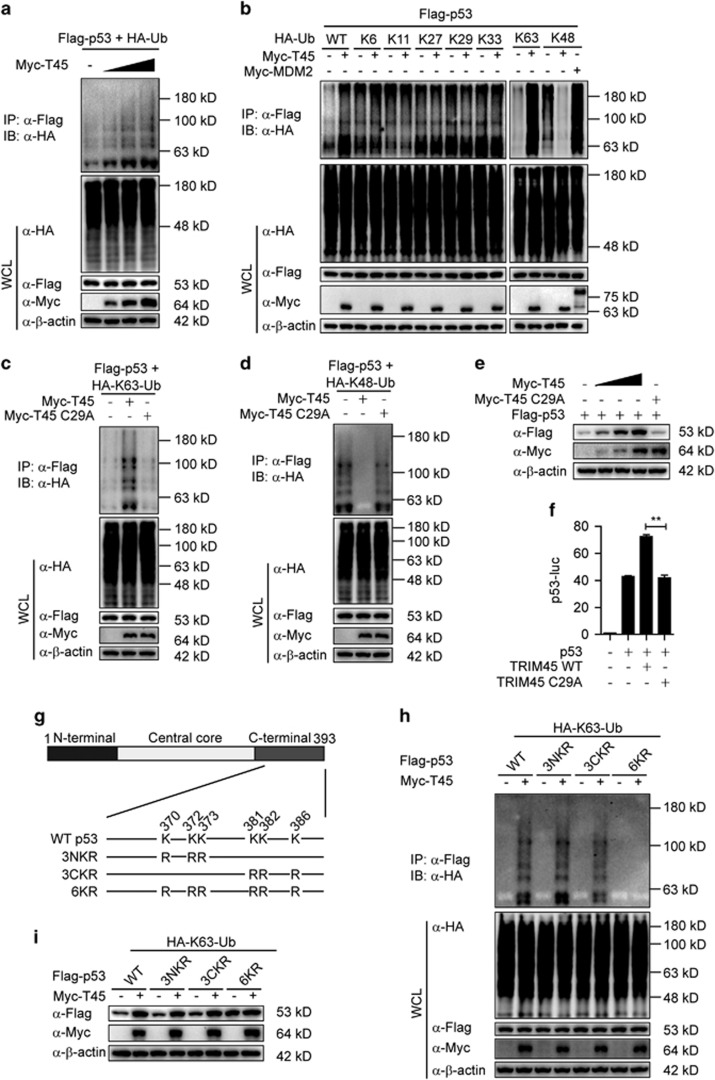
TRIM45 mediates the K48-K63 ubiquitination transition of p53. (**a**) Lysates of U87 cells transfected with plasmids expressing Flag-p53, hemagglutinin (HA)-ubiquitin and increasing amounts of Myc-TIRM45 and treated with MG132 were immunoprecipitated with anti-Flag beads and immunoblotted with an anti-HA antibody. (**b**) U87 cells were transfected with plasmids expressing Flag-p53, Myc-TIRM45 or Myc-MDM2 together with HA-ubiquitin or its indicated mutants in the presence of MG132. The cell lysates were immunoprecipitated with anti-Flag beads and immunoblotted with an anti-HA antibody. (**c** and **d**) Lysates of U87 cells transfected with plasmids expressing Flag-p53, HA-tagged K63-linked ubiquitin (HA-K63-Ub) (**c**) or HA-K48-Ub, (**d**) together with Myc-TIRM45 or Myc-TIRM45 C29A and treated with MG132 were immunoprecipitated with anti-Flag beads and immunoblotted with anti-HA antibody. (**e**) Immunoblot analysis of extracts of U87 cells transfected with Flag-p53 and increasing amounts of HA-TRIM45 or HA-TRIM45 CA (**f**) Luciferase activity in U87 cells transfected with p53-luc, p53, and Myc-TIRM45 or Myc-TRIM45 C29A. Results are presented as Firefly luciferase activity relative to *Renilla* luciferase activity. Each bar represents the mean±S.D. of three independent experiments. **P<0.01. (**g**) A schematic diagram of p53 mutants. (**h**) Lysates of U87 cells transfected with plasmids expressing HA-K63-Ub and wild-type (WT) p53 or p53 mutant together with Myc-TRIM45 or empty vector and treated with MG132 were immunoprecipitated with anti-Flag beads and immunoblotted with an anti-HA antibody. (**i**) Immunoblot analysis of extracts of U87 cells transfected with plasmids expressing HA-K63-Ub and WT p53 or p53 mutant together with Myc-TRIM45 or empty vector

As *TRIM45* is an E3 ligase,^19^ we next determined whether *TRIM45* affects p53 stability via ubiquitination. First, we found that *TRIM45* promoted the polyubiquitination of p53 in a dose-dependent manner (Figure 6a). Having demonstrated that *TRIM45* is a p53-interacting E3 ligase that targets p53 for ubiquitination, we further characterized the specific types of polyubiquitination of p53 by TRIM45. Interestingly, we found that *TRIM45* promoted the K63-linked ubiquitination and inhibits the K48-linked ubiquitination of p53, but did not affect other ubiquitination types of p53 (Figure 6b). As a positive control, MDM2 promoted the K48-linked ubiquitination of p53 (Figure 6b). To assess the possible role of MDM2 in TRIM45-mediated effect on p53, we tested whether *TRIM45* could affect p53 ubiquitination in MDM2-knockdown U87 cells. We found that MDM2-knockdown significantly enhanced the TRIM45-mediated K63-linked ubiquitination of p53. However, *TRIM45* overexpression could not inhibit the K48-linked ubiquitination of p53 in MDM2-knockdown cells (Supplementary Figure 10). We next investigated whether *TRIM45* inhibits K48-linked ubiquitination through disrupting the MDM2–p53 complex. The co-IP assay showed that *TRIM45* did not affect the interaction between MDM2 and p53 (Supplementary Figure 11). Consistent with the results in Figure 6b, mutation of K48 to arginine (Ub-K48R) markedly increased its ability to be conjugated to p53 by TRIM45, while Ub-K63R reduced its ability to be conjugated to p53 by *TRIM45* (Supplementary Figure 12). Furthermore, *TRIM45* overexpression potentiated the total ubiquitination and K63-linked ubiquitination and inhibited the K48-linked ubiquitination of endogenous p53 (Supplementary Figure 13). These data suggest that *TRIM45* mediates the K48-K63 ubiquitination transition of p53.

We next investigated whether *TRIM45* mediates the K48-K63 ubiquitination transition of p53 via its E3 ligase activity, and found that the *TRIM45* mutant C29A that lacks E3 ligase activity failed to promote the K63-linked ubiquitination or inhibit the K48-linked ubiquitination of p53 in the presence of MG132 (Figures 6c and d). In addition, wild-type TRIM45, but not *TRIM45* C29A, increased p53 protein levels (Figure 6e). Consistent with this result, *TRIM45* C29A did not affect the transcriptional activity of p53 (Figure 6f). Taken together, these results indicate that the E3 ligase activity of *TRIM45* is indispensable for the K48-K63 ubiquitination transition of p53 and for p53 stabilization and activation.

### *TRIM45* stabilizes p53 by catalyzing the K63-linked polyubiquitination of p53 at its C-terminal six lysine residues

**Figure 7:**
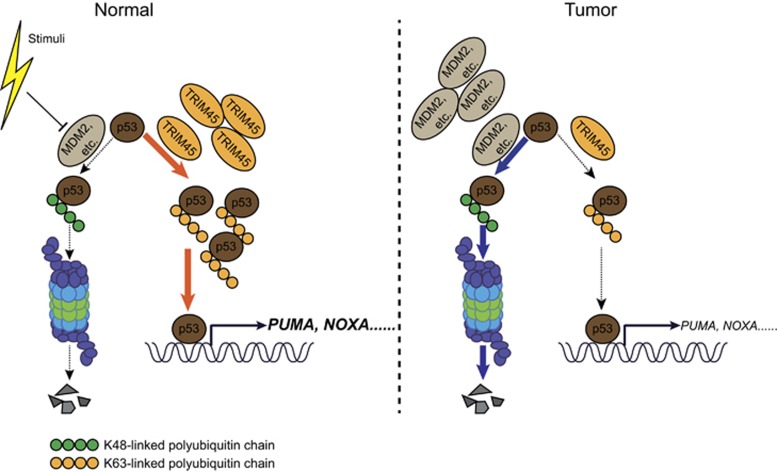
A proposed model illustrating the mechanism by which *TRIM45* regulates p53 stability

K48-linked polyubiquitination has a critical role in p53 degradation, and the major lysine residues that are ubiquitinated by E3 ligases are the six lysines in the C-terminal of p53, including K370, K372, K373, K381, K382, and K386.^23^ The results presented in Figures 6b–d prompted us to speculate that TRIM45-mediated K63-linked polyubiquitination might compete with K48-linked polyubiquitination at the same lysine residues, thereby protecting p53 from K48-linked polyubiquitination. To test this hypothesis, we constructed a series of Flag-tagged p53 mutants with multiple lysine residues substituted with arginine (R) residues (Figure 6g). We found that the K63-linked ubiquitination mediated by *TRIM45* was nearly abolished in the p53 6KR mutant but not in other p53 mutants (Figure 6h and Supplementary Figure 14). Consistent with this observation, *TRIM45* failed to stabilize p53 6KR, but was still able to stabilize the other p53 mutants (Figure 6i). These data suggest that *TRIM45* mainly mediates K63-linked polyubiquitination on the C-terminal six lysine residues of p53, thereby inhibiting the availability of those residues for the subsequent K48-linked polyubiquitination that targets p53 for degradation (Figure 7).

## Discussion

The p53 tumor suppressor network is frequently disrupted in GBM, and the deregulation of p53 antagonists, such as MDM2 and MDM4 (also known as HDMX and MDMX), is a primary contributor to p53 inactivation in this context.^24^ The MDM2 proteins are dysregulated in many human cancers, and they exert their oncogenic activity predominantly by inhibiting the activity of p53. High MDM protein levels can result from gene amplification, increased transcription and aberrant PTMs.^24^ MDM2 is the primary E3 ubiquitin ligase responsible for the conjugation of K48-linked polyubiquitin chains to p53 for its degradation. In addition to MDM2, other molecules, including Pirh2, COP1, and ARF-BP1 also conjugate K48-linked polyubiquitin chains to p53 to target it for proteasomal degradation.^25, 26, 27^ These E3 ligases might have redundant functions in regulating p53 degradation.

In contrast to the aforementioned E3 ligases that mediate p53 degradation, several E3 ligases can also stabilize p53 or promote p53 transcriptional activity. WWP1, an HECT domain E3 ligase, induces p53 ubiquitination and stabilizes p53.^28^ Interestingly, Ubc13, a key E2 ubiquitin-conjugating enzyme that induces K63-linked ubiquitination, also promotes K63-linked ubiquitination of p53, and protects p53 from degradation.^29^ In the current study, we found that *TRIM45* regulates p53 stability using a similar mechanism. *TRIM45* can promote K63-linked polyubiquitination and inhibit K48-linked polyubiquitination of p53 (Figure 6b). It is tempting to speculate that *TRIM45* acts in conjunction with the E2 ligase Ubc13 to conjugate K63-linked polyubiquitin chains to p53.

Polyubiquitination of p53 catalyzed by *TRIM45* was mapped to C-terminal six lysine residues (Figure 6h and Supplementary Figure S8), which are also targeted by MDM2 for K48-linked polyubiquitination.^30, 31^ These findings suggest that the polyubiquitin chains of distinct linkages may compete for the same residues of p53. Different modifications by different regulators are known to compete with each other for the same site in some proteins. Direct competition between acetylation and ubiquitination for the same C-terminal lysine residues of p53 has been reported to stabilize and activate p53.^32, 33^ It has also been reported that the competition between K11- and K48-linked polyubiquitination stabilizes a critical antiviral regulator, STING.^34^

Taken together, these data support a model in which *TRIM45* functions as a tumor suppressor by stabilizing p53 (Figure 7). In normal cells, p53 protein amount is tightly controlled by MDM2, or other E3 ligases that catalyze the K48-linked polyubiquitination of p53 for proteasome-dependent degradation. Meanwhile, *TRIM45* can compete with MDM2 or other E3 ligases to promote the K63-linked polyubiquitination of p53, thereby preventing the K48-linked polyubiquitination of p53 and protecting it from degradation. This process may let cells undergo apoptosis under harmful stresses. In glioma tissues, *TRIM45* is downregulated and cannot maintain the sufficient amount of p53 to induce the apoptosis of tumor cells.^35^ Our current findings highlight a previously undescribed mechanism for the regulation of p53 stability.

In summary, we have demonstrated that *TRIM45* promotes the K63-linked ubiquitination of p53 to protect p53 from degradation and inactivation, thereby suppressing GBM proliferation and tumorigenicity. These results suggest that *TRIM45* acts as a tumor suppressor in malignant glioma. A more comprehensive understanding of the role *TRIM45* has in the pathogenesis of malignant glioma may provide an opportunity to develop a novel therapeutic strategy by restoration of *TRIM45* expression.

## Materials and methods

### Clinical specimens

The samples were obtained from 40 glioma patients who had undergone neurosurgery between 2008 and 2013 at the Third Affiliated Hospital of Soochow University. All samples were obtained from newly diagnosed patients with histologically confirmed primary gliomas. The pathological diagnosis and grading of glioma were assigned according to the WHO classification system. The normal brain tissues were obtained from individuals who had died in traffic accidents and were confirmed to be free of any prior pathologically detectable conditions. Taken together, the collection of samples used in this study comprised normal brain tissue (*n*=10), pilocytic astrocytoma (WHO grade I; *n*=4), diffuse astrocytoma (WHO grade II; *n*=7), anaplastic astrocytoma (WHO grade III; *n*=12), and GBM (WHO grade IV; *n*=17) tissues. The study was approved by the Research Ethics Board of the Third Affiliated Hospital of Soochow University. Informed consent was obtained from all individual participants included in the study.

### Cell culture and transfection

U87 and LN229 cells were cultured in DMEM (Life Technologies, Beijing, China) supplemented with 10% fetal bovine serum (Gibco, Grand Island, NY, USA) and 1 mM glutamine (Life Technologies). For the gene knockdown and KO assays, cells were infected with lentivirus encoding shRNA or sgRNA, respectively. For the gene overexpression assays, cells were transfected with lentivirus encoding the *TRIM45* coding sequence.

### Plasmids and antibodies

The *TRIM45* expression plasmid was constructed using the GATEWAY System (Life Technologies, Carlsbad, CA, USA). The HA-tagged p53 expression plasmid and the p53 domain deletion mutants were kindly provided by Dr. Tiebang Kang (Sun Yat-sen University, Guangzhou, China). All mutants were generated using standard molecular techniques. The anti-TRIM45 antibody (NBP1-53109) was purchased from Novus (Littleton, CO, USA) and anti-p53 (sc-126) was obtained from Santa Cruz (Santa Cruz, CA, USA). Horseradish peroxidase (HRP)-anti-Flag (M2) (A8592) and anti-*β*-actin (A1978) were purchased from Sigma (St. Louis, MO, USA). HRP-anti-hemagglutinin (12013819001) and anti-c-Myc-HRP (11814150001) were purchased from Roche Applied Science (Mannheim, Germany). The anti-caspase-3 (25546-1-AP) was purchased from Proteintech (Wuhan, Hubei, P.R.C). The anti-MDM2 (YT5186) was purchased from ImmunoWay (Newark, DE, USA). The anti-K63-linked ubiquitin antibody (05-1308) was obtained from Millipore (Billerica, MA, USA). The anti-phospho-Akt (no. 4060), anti-Akt (no. 4691), anti-phospho-JNK (no. 9251), anti-JNK (no. 9252), anti-phospho-ERK (no. 9101), anti-ERK (no. 9102), anti-phospho-p38 (no. 9211), anti-p38 (no. 9212), anti-Puma (no. 4976), and anti-K48-linked ubiquitin (no. 4289) antibodies were obtained from Cell Signaling Technology (Danvers, MA, USA).

### Generation of *TRIM45* KO cells by CRISPR/Cas9 technology

CRISPR/Cas9 system-mediated KO was performed as described previously,^36^ and the sequence of the target GFP or *TRIM45*–gRNA are as follows: GFP sgRNA, 5′-GGGCGAGGAGCTGTTCACCG-3′, *TRIM45* sgRNA 1, 5′-GATGCTGGAGAGCCTACGTG-3′, and *TRIM45* sgRNA 2, 5′-GTGTGACCTGTGCAACGACA-3′.

### IP and immunoblot analysis

Cells were extracted in ice-cold low-salt lysis buffer (50 mM HEPES, 150 mM NaCl, 1 mM EDTA, 1.5 mM MgCl_2_, 10% glycerol, 1% Triton X-100) supplemented with 5 mg/ml protease inhibitor cocktail (Roche). Protein concentration was measured with BCA Protein Assay Kit (Pierce, Rockford, IL, USA). For IP experiments, whole-cell extracts were prepared after transfection, followed by incubation overnight with the appropriate antibodies and Protein A/G beads (Thermo, Rockford, IL, USA) or anti-Flag/anti-HA beads (Sigma-Aldrich, St. louis, MO, USA). Beads were washed three times with low-salt lysis buffer. Immunoprecipitates were eluted with 3 × SDS Loading Buffer and resolved by SDS-PAGE. Proteins were transferred to PVDF membranes (Bio-Rad, Hercules, CA, USA) and incubated with the appropriate antibodies. For the ubiquitin assays, cell lysates were first immunoprecipitated with anti-Flag beads, then boiled in the presence of 1% SDS, followed by a second IP with anti-Flag, so that only polyubiquitination modification would be detected by immunoblot analysis with anti-hemagglutinin.

### Luciferase assay

U87 cells were plated in 24-well plates and transfected with plasmids expressing p53-luc (50 ng) and TK-Renilla-luc (10 ng), together with 200 ng Flag-tagged p53 and increasing amounts of *TRIM45* using Lipofectamine 2000 (Life Technologies). An empty pcDNA3.1 vector was used to maintain equal amounts of DNA between wells. Cells were harvested 24 h post transfection, and luciferase activities were measured with Dual-luciferase Assay Kits (Promega, Madison, WI, USA) according to the manufacturer’s protocol. Reporter gene activity was determined by normalizing Firefly luciferase to Renilla luciferase activity.

### Cell proliferation and anchorage-independent growth assays

Cell proliferation was measured by directly counting the number of the cells. Briefly, triplicate plates of cells were trypsinized and stained with Trypan blue, and unstained cells were counted using a hemocytometer. Anchorage-independent growth assays were performed in 6-well plates. Cell were seeded at a density of 1 × 10^4^ cells per well in DMEM+10% FBS containing 0.35% low-melting agarose on top of the agar layer containing 0.5% low-melting agarose DMEM+10% FBS. Colonies were counted 14–21 days after seeding.

### Xenograft tumor formation in mice

Xenograft tumor formation assays were conducted as described previously.^37^ Briefly, BALB/c-nude mice (4-week-old) were purchased from Guangdong Medical Laboratory Animal Center. All experimental procedures were approved by the Institutional Animal Care and Use Committee of the Sun Yat-sen University. The mice were subcutaneously inoculated with 5 × 10^6^ of the indicated cells. Tumor growth was monitored every five days. Tumor volume was calculated using the formula: *V*=0.5 × length × width × width. The tumor-bearing mice were killed 40 days after inoculation, and the tumors were subsequently removed for further study.

### Annexin V-binding assay

The FITC-Annexin V Apoptosis Detection Kit I (BD Bioscience, Franklin Lakes, NJ, USA) was used to quantify apoptotic cells according to the manufacturer’s instructions.

### Statistical analysis

The data are represented as the mean±S.D. when indicated, and the Student’s *t*-test was used for all statistical analyses using the GraphPad Prism 5.0 software (GraphPad Software, Inc., La Jolla, CA, USA). Differences between groups were considered statistically significant when the *P*-value was <0.05.
