# Supplementary material for: TRIM45 functions as a tumor suppressor in the brain via its E3 ligase activity by stabilizing p53 through K63-linked ubiquitination
Source: Cell Death Dis. 2017 May 25;8(5):e2831–. doi: 10.1038/cddis.2017.149 (PMC5520693; doi:10.1038/cddis.2017.149)
Supplement: Supplementary Information [file cddis2017149x1.docx]

**Supplementary Information**

TRIM45 functions as a tumor suppressor in the brain via its E3 ligase activity by stabilizing p53 through K63-linked ubiquitination

Jindong Zhang, Chuanxia Zhang, Jun Cui, Jiayu Ou, Jing Han, Yunfei Qin, Feng Zhi, and Rong-Fu Wang

**Supplementary Figures 1-14**

**Supplementary Figure 1**

**
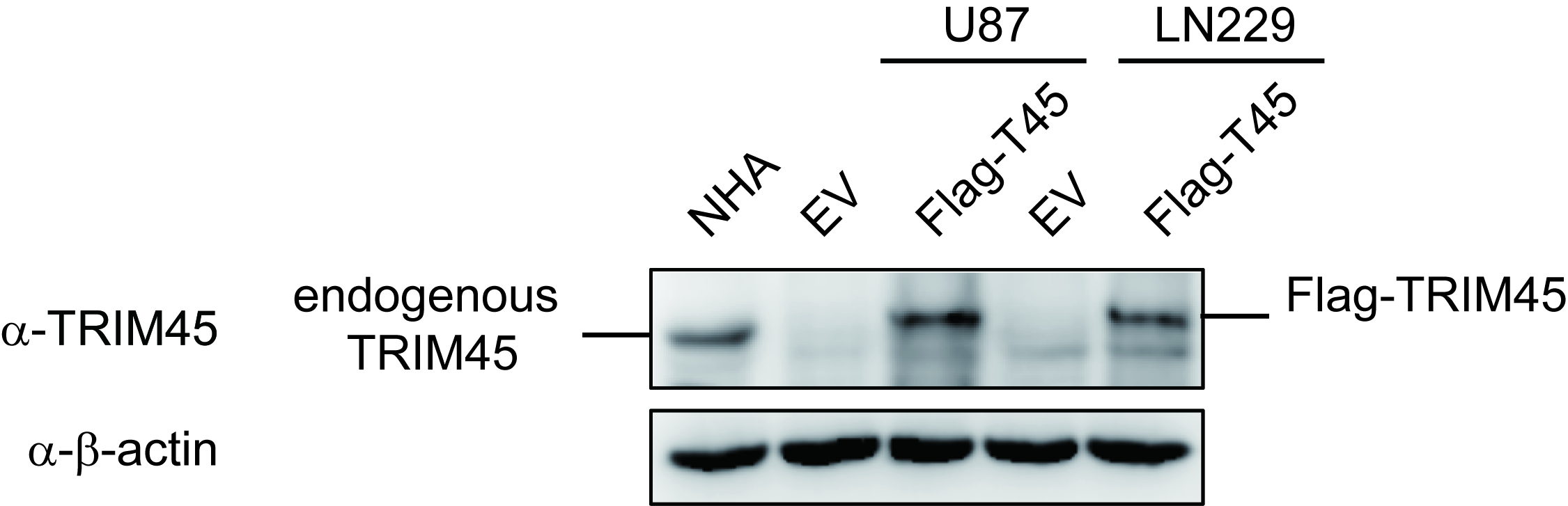
**

**Supplementary Figure 1. Comparable expression levels of Flag-TRIM45 and endogenous TRIM45 genes.** Lysates of primary normal human astrocytes (NHAs) and TRIM45-overexpressing and control U87 and LN229 cells were immunoblotted with anti-TRIM45 antibody. β-actin was used as a loading control.

**Supplementary Figure 2**

**
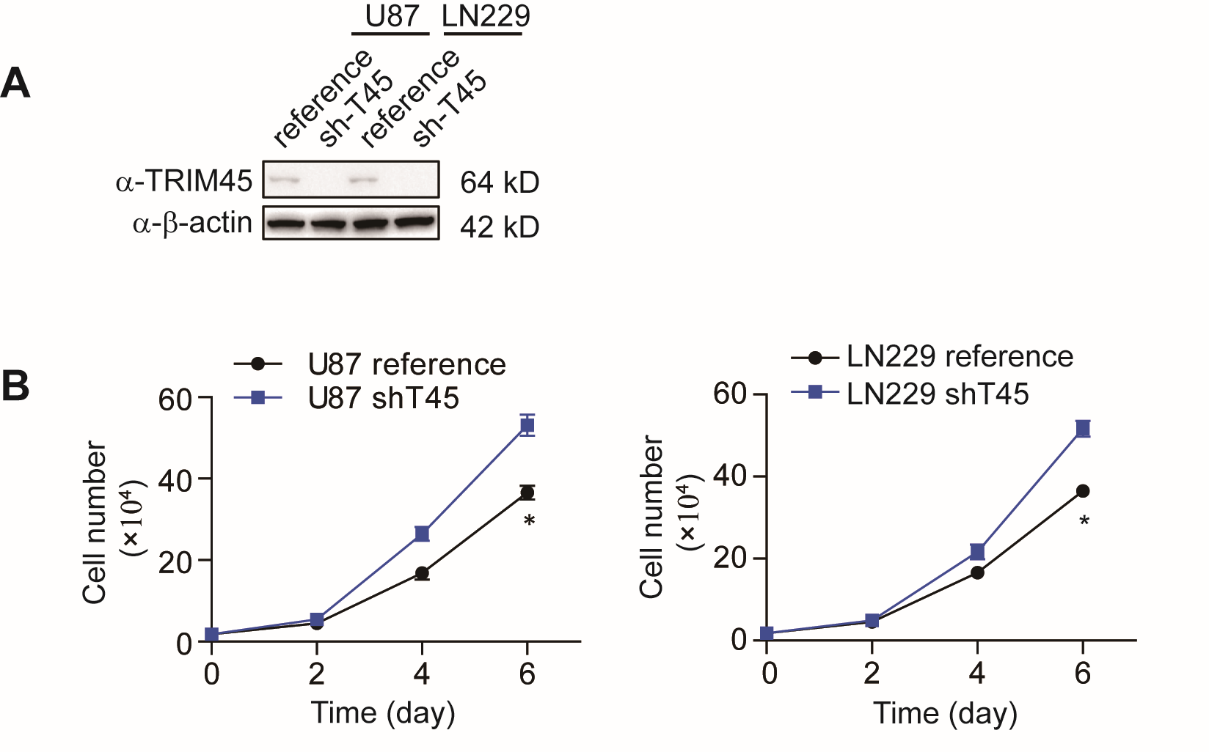
**

**Supplementary Figure 2**. TRIM45 knockdown promotes the proliferation of glioma cells. (A) Lysates of TRIM45 knockdown and control U87 and LN229 cells were immunoblotted with anti-TRIM45 antibody. β-actin was used as a loading control. (B) Proliferation assay of U87 and LN229 cells transduced with TRIM45 specific shRNA (shT45) or reference shRNA. Each bar represents the mean ± S.D. of 3 independent experiments. **P* < 0.05.

**Supplementary Figure 3**

**
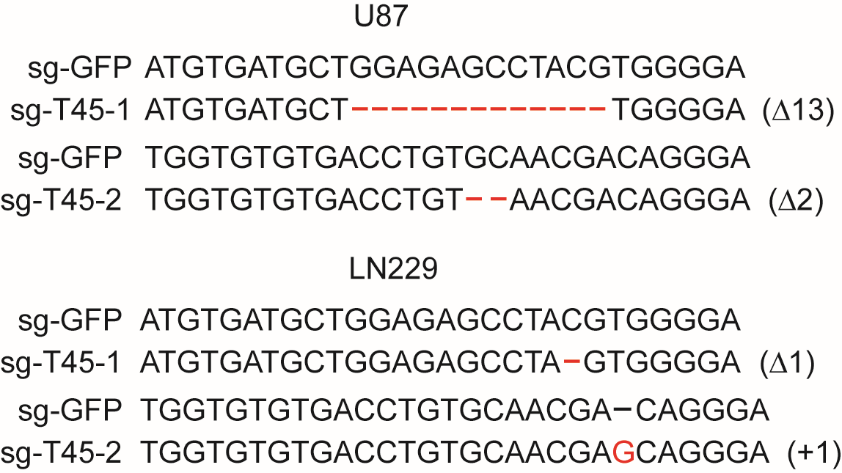
**

**Supplementary Figure 3**. The sequencing results of TRIM45 KO mono-clones.

**Supplementary Figure 4**

**
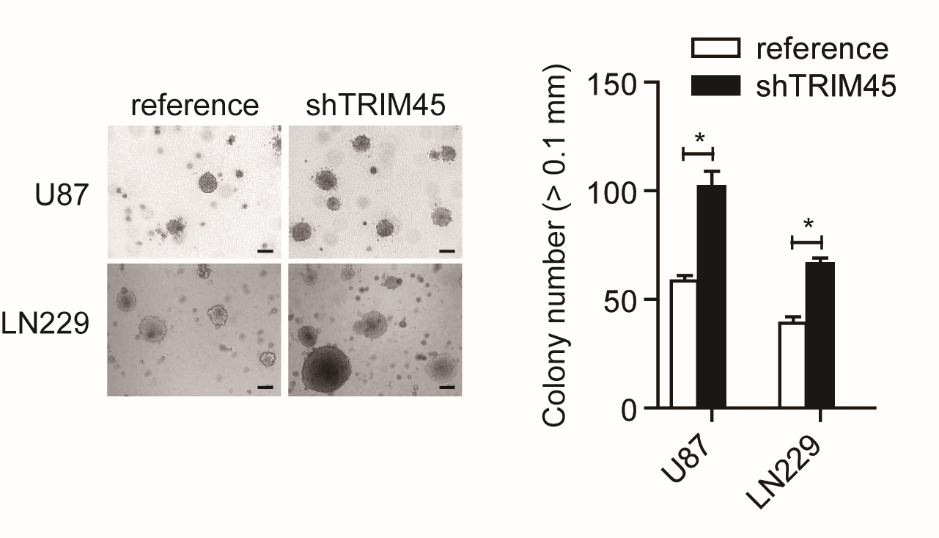
**

**Supplementary Figure 4**. Knockdown of TRIM45 promotes both the number and size of the colonies in both U87 and LN229 cells. Anchorage-independent growth assay of U87 and LN229 cells transduced with TRIM45-targeting shRNA (shTRIM45) or reference shRNA. Scale bars: 200 μm. Each bar represents the mean ± S.D. of 3 independent experiments. **P* < 0.05.

**Supplementary Figure 5**

**
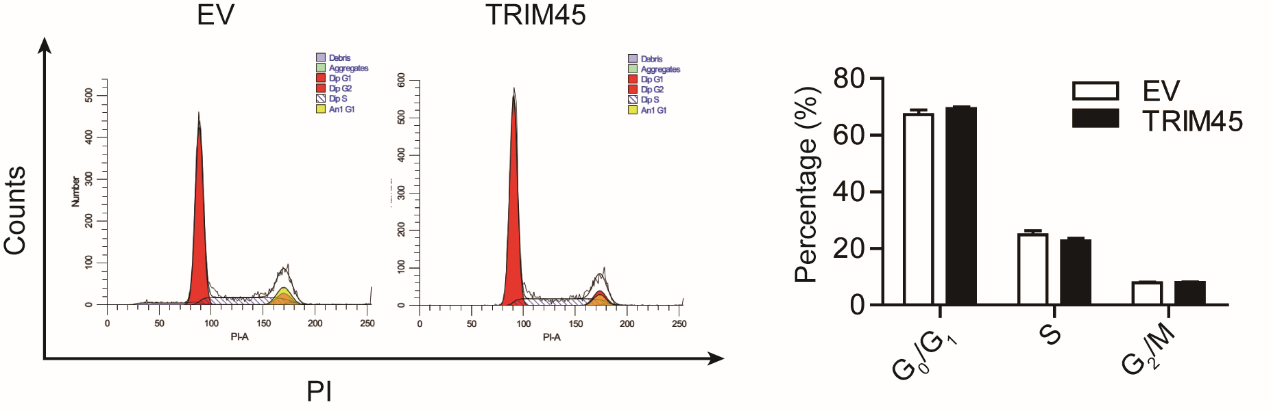
**

**Supplementary Figure 5**. Overexpression of TRIM45 has little effect of cell-cycle distribution. Flow cytometric analysis with propidium iodide staining of TRIM45-overexpressing or control cells. Each bar represents the mean ± S.D. of 3 independent experiments.

**Supplementary Figure 6**

**
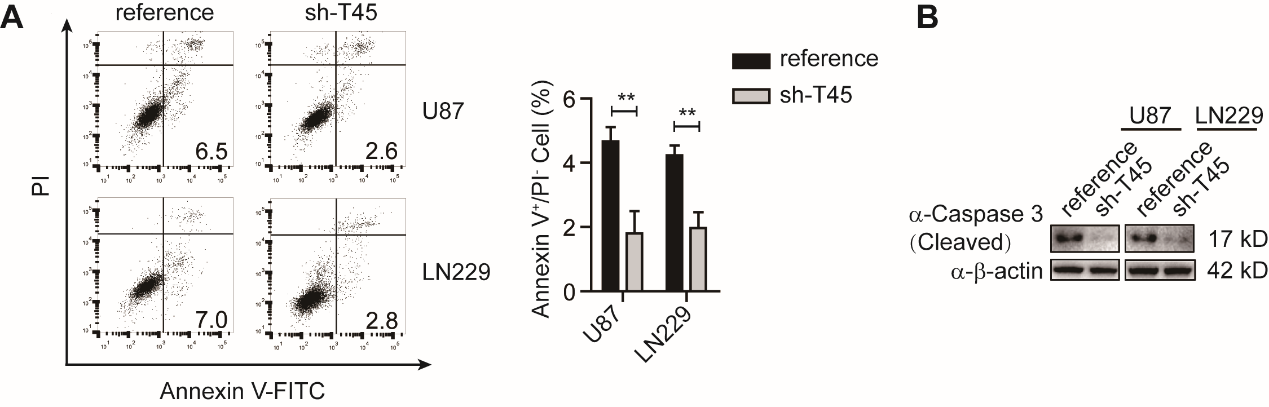
**

**Supplementary Figure 6**. Knockdown of TRIM45 inhibits apoptosis in U87 and LN229 cells. (A) The cells were incubated with FITC-Annexin V solution. The cells were then subjected to FACS analysis to assess the fraction of apoptotic cells. Each bar represents the mean ± S.D. of 3 independent experiments. ***P* < 0.01. (B) Lysates of TRIM45 knockdown and control U87 and LN229 cells were immunoblotted with anti-caspase 3 antibody. β-actin was used as a loading control.

**Supplementary Figure 7**

**
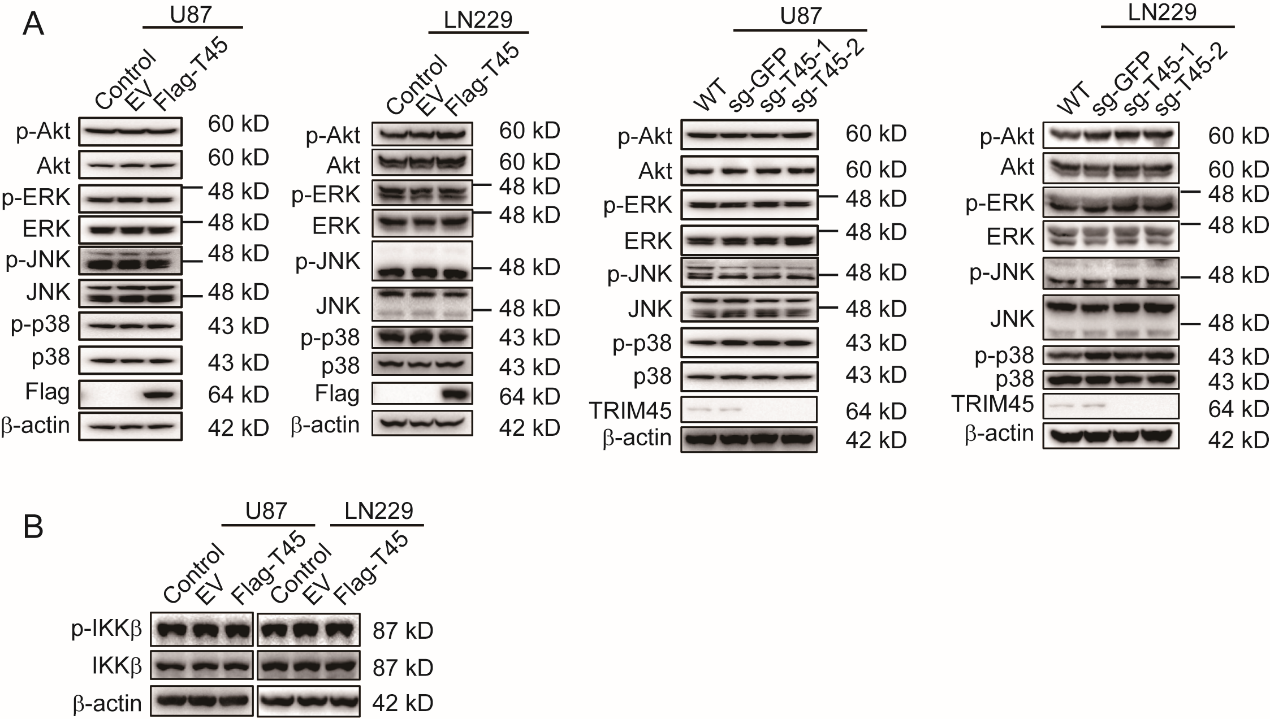
**

**Supplementary Figure 7**. TRIM45 does not affect the MAPK, NF-κB, and PI3K/AKT signaling pathways in glioma cells**.** (A) Immunoblot analysis of p-Akt, total Akt, p-ERK, total ERK, p-JNK, total JNK, p-p38 and total p38 in TRIM45-overexpressing and TRIM45 knockout U87 and LN229 cells. β-actin was used as a loading control. (B) Immunoblot analysis of p-IKKβ and total IKKβ in TRIM45-overexpressing and wild type U87 and LN229 cells. β-actin was used as a loading control.

**Supplementary Figure 8**


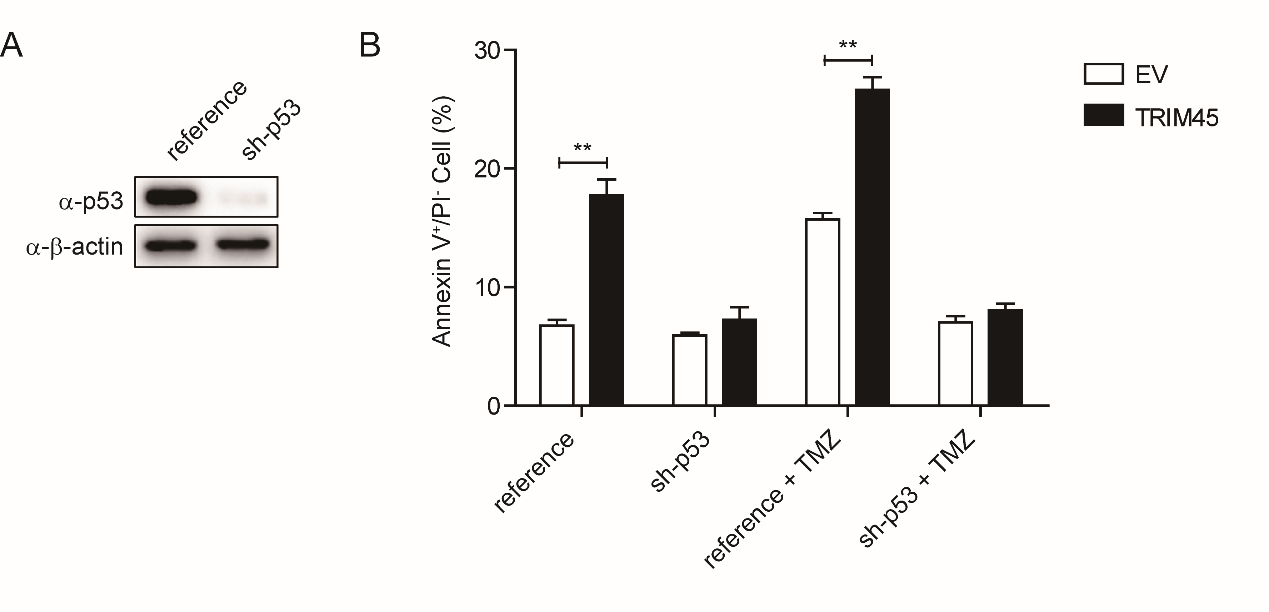


**Supplementary Figure 8**. TRIM45 promotes apoptosis in a p53-dependent manner. (A) Lysates of U87 cells transfected with p53-taggeted shRNA (sh-p53) or reference shRNA (reference) were immunoblotted with anti-p53 antibody. β-actin was used as a loading control. (B) The TRIM45-overexpressing U87 cells were transfected with p53-taggeted shRNA (sh-p53) or reference shRNA (reference) and treated with TMZ or left untreated, then incubated with FITC-Annexin V/PI solution and subjected to FACS analysis.

**Supplementary Figure 9**

**
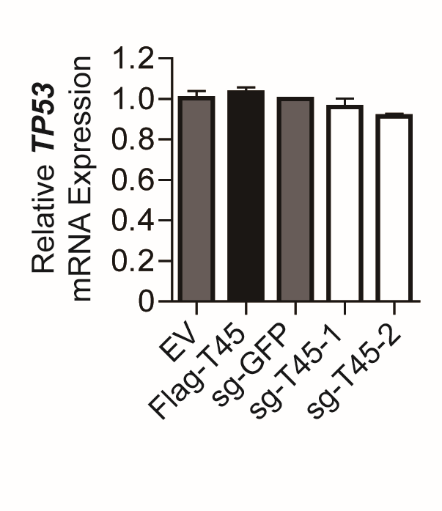
**

**Supplementary Figure 9**. The mRNA levels of p53 target genes in TRIM45 overexpression and knockout U87 cells were analyzed using real-time PCR

**Supplementary Figure 10**

**
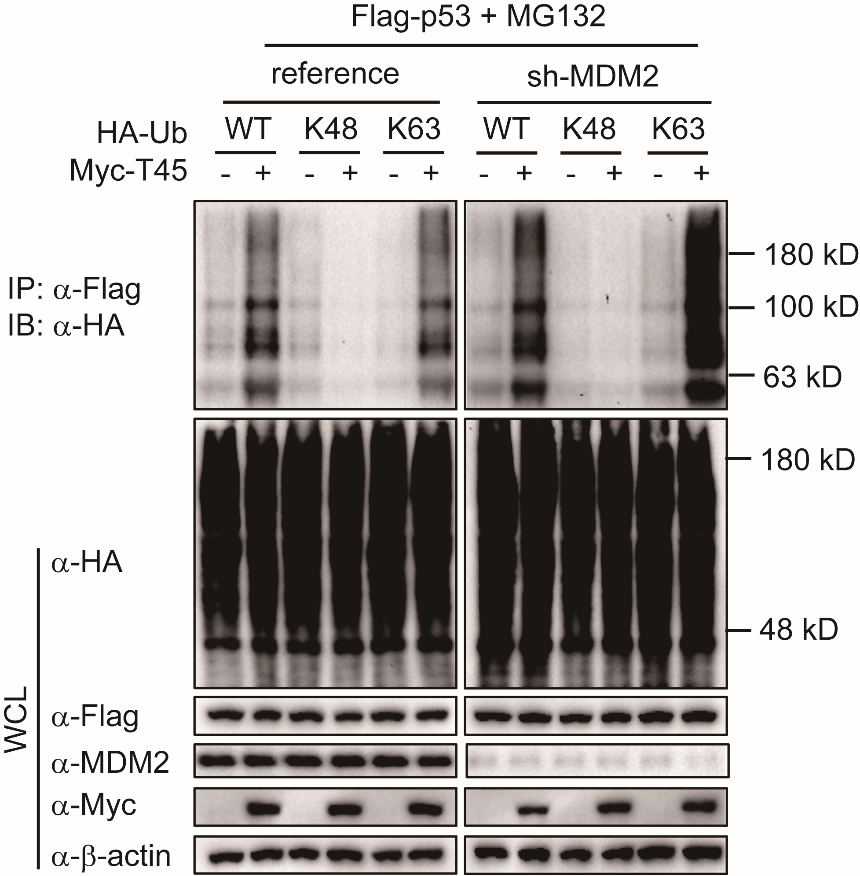
**

**Supplementary Figure 10. Ubiquitination of p53 with or without MDM2 expression.** Lysates of U87 cells transfected with MDM2-specific shRNA or reference shRNA and Flag-p53, Myc-TIRM45 or empty vector together with HA-ubiquitin or its mutants in the presence of MG132 were immunoprecipitated with anti-Flag beads and immunoblotted with anti-HA antibody.

**Supplementary Figure 11**


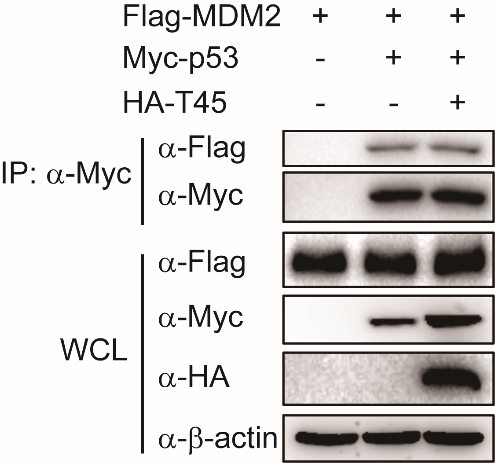


**Supplementary Figure 11.** TRIM45 does not disrupt the interaction between p53 and MDM2. U87 cells were transfected with Flag-MDM2, Myc-p53 and HA-TRIM45 plasmids. The lysates were immunoprecipitated with anti-Myc beads and immunoblotted with anti-Flag antibody.

**Supplementary Figure 12**


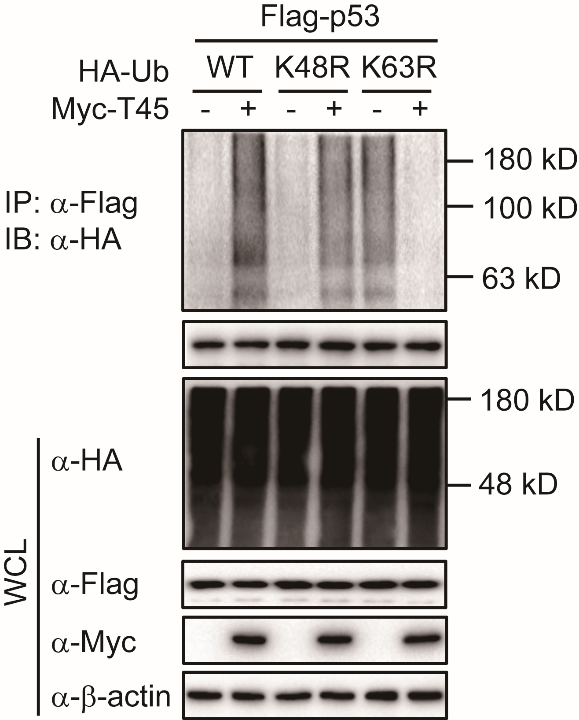


**Supplementary Figure 12**. **Ubiquitination analysis of p53**. U87 cells were transfected with plasmids for Flag-p53, Myc-TIRM45 or empty vector together with HA-ubiquitin or its mutants in the presence of MG132. The cell lysates were immunoprecipitated with anti-Flag beads and immunoblotted with anti-HA antibody.

**Supplementary Figure 13**

**
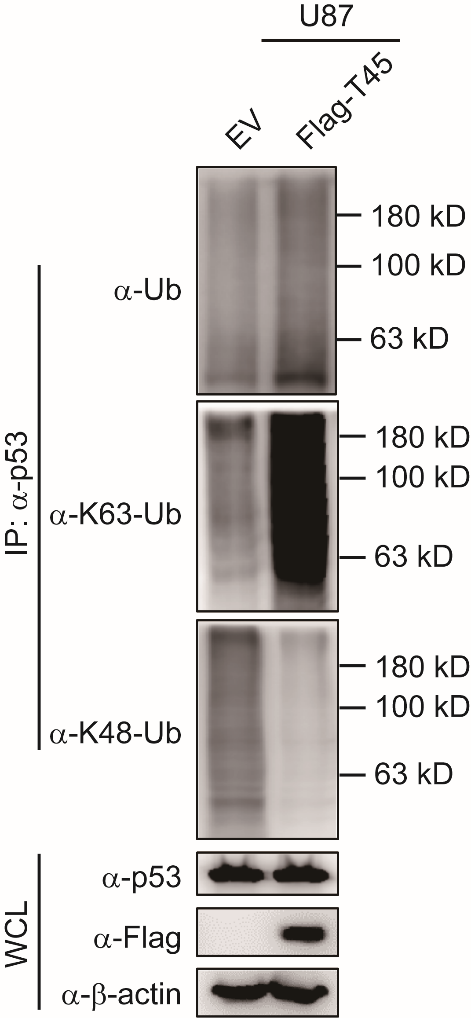
**

**Supplementary Figure 13**. **K48- and K63-linked ubiquitination of p53 in U87 cells with or without ectopic expression of TRIM45.** TRIM45-overexpressing and control U87 cells were pretreated with MG132, immunoprecipitated with anti-p53 antibody and protein G beads and immunoblotted with anti-ubiquitin (wild type), anti-K63 and anti-K48 ubiquitin antibodies.

**Supplementary Figure 14**

**
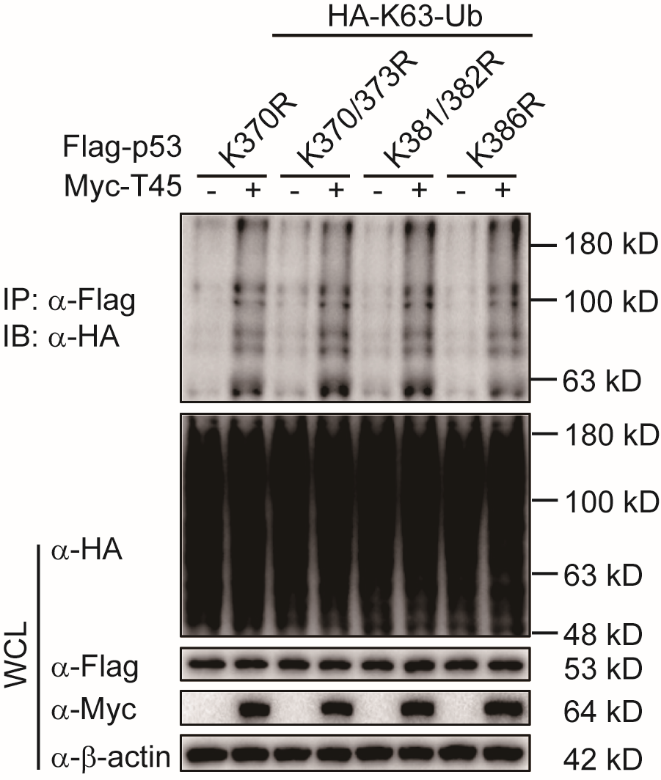
**

**Supplementary Figure 14**. **Ubiquitination analysis in p53 by TRIM45**. TRIM45 catalyzed K63-linked poly-ubiquitination of p53 at its C-terminal six lysine residues. Lysates of U87 cells transfected with plasmids expressing HA-K63-Ub and p53 mutant constructs together with Myc-TIRM45 or an empty vector were treated with MG132 and immunoprecipitated with anti-Flag beads and immunoblotted with an anti-HA antibody.
